# Therapy of pancreatic cancer via an EphA2 receptor-targeted delivery of gemcitabine

**DOI:** 10.18632/oncotarget.7931

**Published:** 2016-03-05

**Authors:** Bridget A. Quinn, Si Wang, Elisa Barile, Swadesh K. Das, Luni Emdad, Devanand Sarkar, Surya K. De, Susan Morvaridi Kharagh, John L. Stebbins, Stephen J. Pandol, Paul B. Fisher, Maurizio Pellecchia

**Affiliations:** ^1^ Department of Human and Molecular Genetics, VCU Institute of Molecular Medicine and VCU Massey Cancer Center, Virginia Commonwealth University, School of Medicine, Richmond, VA 23298, USA; ^2^ Sanford-Burnham-Prebys Medical Discovery Institute, La Jolla, CA 92037, USA; ^3^ Division of Biomedical Sciences, School of Medicine, University of California Riverside, Riverside, CA 92521, USA; ^4^ Department of Medicine, Cedars-Sinai Medical Center, Los Angeles, CA 90048, USA

**Keywords:** 123B9, EphA2, targeted delivery, drug-conjugates, gemcitabine

## Abstract

First line treatment for pancreatic cancer consists of surgical resection, if possible, and a subsequent course of chemotherapy using the nucleoside analogue gemcitabine. In some patients, an active transport mechanism allows gemcitabine to enter efficiently into the tumor cells, resulting in a significant clinical benefit. However, in most patients, low expression of gemcitabine transporters limits the efficacy of the drug to marginal levels, and patients need frequent administration of the drug at high doses, significantly increasing systemic drug toxicity. In this article we focus on a novel targeted delivery approach for gemcitabine consisting of conjugating the drug with an EphA2 targeting agent. We show that the EphA2 receptor is highly expressed in pancreatic cancers, and accordingly, the drug-conjugate is more effective than gemcitabine alone in targeting pancreatic tumors. Our preliminary observations suggest that this approach may provide a general benefit to pancreatic cancer patients and offers a comprehensive strategy for enhancing delivery of diverse therapeutic agents to a wide range of cancers overexpressing EphA2, thereby potentially reducing toxicity while enhancing therapeutic efficacy.

## INTRODUCTION

Pancreatic cancer is an extremely aggressive and deadly disease, with a 5-year survival rate of less than 5%. Most tumors are either locally advanced or have metastasized at the time of diagnosis and, intrinsically, this cancer is extremely resistant to chemotherapy and radiation. Currently, first line treatment for pancreatic cancer consists of surgical resection, if possible, and a subsequent course of chemotherapy [1]. This chemotherapy usually consists of treatment with Gemcitabine [2].

In 1997, Burris, et al. published a clinical study comparing Gemcitabine to 5-Fluoruracil (5-FU) for the treatment of pancreatic cancer [3]. In this study, 126 patients were enrolled with 63 per treatment group. 23.8% of patients showed clinical benefit with Gemcitabine, as compared to only 4.8% of 5-FU-treated patients. The median survival was 5.65 months for Gemcitabine and 4.41 months for 5-FU. Finally, 18% of patients treated with Gemcitabine were alive at a 12-month time point, while survival at this time point for patients treated with 5-FU was only 2% [3]. This trial encouraged the FDA to approve Gemcitabine for the treatment of pancreatic cancer in 1998. Gemcitabine is currently a standard treatment used for patients with pancreatic cancer, although the drug only provides minimal benefit to patients. Gemcitabine triphosphate acts as an analog for deoxycytidine triphosphate, which allows it to be incorporated into DNA during replication. After Gemcitabine is incorporated, another nucleotide may be added to the chain, but inhibition of chain elongation subsequently occurs. DNA damage repair is not able to remove the drug and, consequently, apoptosis occurs [4].

Gemcitabine enters the cell through multiple cell membrane transporters, although the sodium-independent transporter, hENT1, has been shown to preferentially transport Gemcitabine [4]. Though there are multiple mechanisms of Gemcitabine resistance, one important mechanism revolves around expression of this protein. Giovannetti et al. showed that patients with tumors that express high amounts of hENT1 have a greater survival advantage with Gemcitabine treatment as compared to those with lower hENT1 expression [5]. Patients with higher hENT1 expression have tumors that can more readily take up Gemcitabine, leading to an increased clinical benefit. However, in many tumors, low expression of Gemcitabine transporters translates into a need for the drug to be administered frequently and at high doses, significantly increasing systemic drug toxicity.

Because systemic toxicity and drug delivery are major issues with most chemotherapeutic agents, the creation of targeted therapies that lower the risk of toxicity has become an attractive strategy in developing novel cancer therapeutics. Targeted therapies focus on attacking cancer cells specifically while sparing normal cells to reduce side effects. One specific strategy of targeted therapy involves modifying currently used drugs to make them cancer specific. This often involves identifying a biomarker on cancer cells that the modified drug can target. One such target is the EphA2 receptor.

Eph receptors are a family of tyrosine kinase receptors involved in neuronal connectivity, blood vessel development, and cell-cell interactions [6−27]. The receptor sub-type EphA2 was identified in cancer cells where it is often highly expressed, mediating communication not only between individual cancer cells, but also between cancer cells and surrounding stromal or vascular cells [6−27]. Despite EphA2 overexpression, expression of ephrinA1, its ligand, often remains normal even in a cancerous state. This can lead to the accumulation of un-activated EphA2 and subsequent oncogenic activity [6].

Peptides have been developed that, similar to the natural ligand for this receptor, selectively bind EphA2 and cause receptor activation and internalization. Hence, in principle, these peptides can be chemically linked to commonly used chemotherapeutic drugs and act as specific delivery agents for these drugs to tumor cells. Once the receptor is activated, the peptide and its attached drug are internalized via a lysosomal pathway, where the peptide is degraded and the drug is released and free to exert its toxic effects on the cell [28]. Our previous studies have shown that Paclitaxel conjugated with these peptides showed increased efficacy in prostate and renal cancers [28−31]. EphA2-targeting molecules reported thus far include two 12-mer peptides, named YSA (of amino acid sequence YSAYPDSVPMMS) and SWL (of amino acid sequence SWLAYPGAVSYR), that have been identified via phage display techniques [32]. Subsequently, we reported on the agent YNH (of amino acid sequence YSAYPDSVP(*Nle) (Hsr)*S, where *Nle* and *Hsr* represent L-norleucine and L-homo-serine, respectively) [28, 31] and very recently we designed a related EphA2 agonist, named 123B9, that presented increased plasma and *in vivo* stability over YSA and YNH [33]. These agents were designed to specifically bind to the EphA2 receptor ligand binding domain (LBD). We demonstrated that drug conjugates via such agents induce receptor activation and internalization of the drug selectively to EphA2 expressing cancer cells and tumor vasculature [28−31]. In particular, we demonstrated that a targeted delivery strategy of Paclitaxel using these EphA2 targeting agents is more efficacious than Paclitaxel alone in xenograft models of pancreatic cancer [30].

Gemcitabine, as mentioned above, although the current first-line treatment for pancreatic cancer, does not offer great therapeutic benefit to most patients. Hence, the goal of this study was to design and characterize a novel targeted delivery strategy for Gemcitabine by conjugating it with our EphA2 targeting agents. Our experiments clearly suggest that conjugation of Gemcitabine with our EphA2 targeting agents increase its efficacy in animal models. Further studies on the use of EphA2 targeting agents such as YNH or 123B9 to direct chemotherapy to pancreatic cancers is therefore warranted to devise innovative and perhaps more effective therapies for this invariably fatal and aggressive cancer.

## RESULTS

### Design and synthesis of EphA2-targeting agents conjugated with gemcitabine

**Figure 1:**
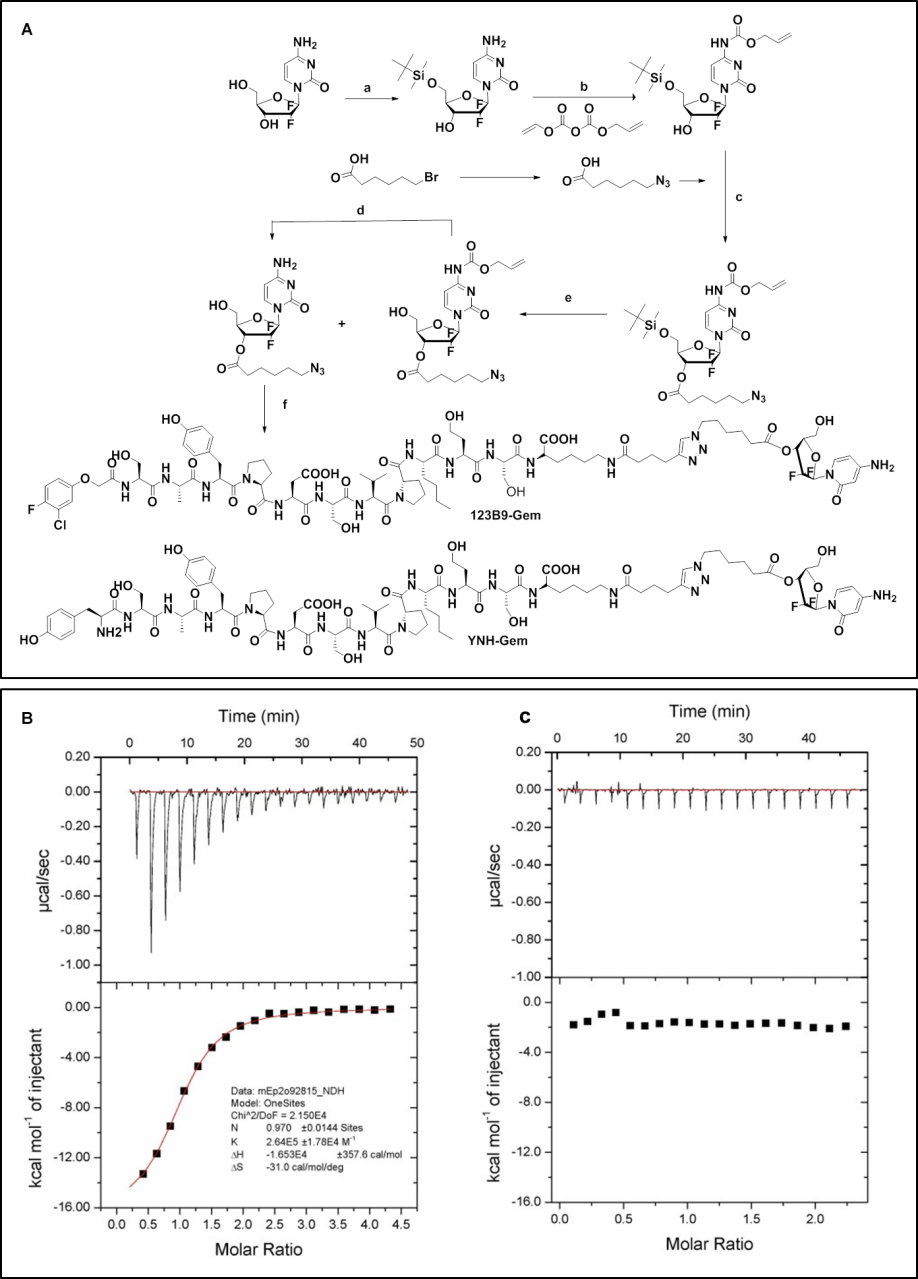
Chemical structures and general synthetic scheme used to obtain the EphA2 targeting agents conjugated with gemcitabine (**A**) Synthetic scheme. Regents and condition: *a* TBSCl, imidazole, rt, 12 h; *b* Diallyl pyrocarbonate, Et3N, THF, rt, 12 h; *c* 6-azido-hexanoic acid, EDCI, DMAP, DCM, rt, 6 h; *d* TBAF, THF, rt, 2 h; *e* (Ph_3_P)_2_PdCl_2_, Bu_3_SnH, HOAc, THF, 0°C, 2 h; *f* YNH motif or 123B9 motif (Supplementary Figure S1), CuSO_4_, sodium ascorbate, DMSO/water, rt, 48 hf) YNH motif or 123B9 motif, CuSO_4_, sodium ascorbate, DMSO/water, rt, 48 h. YDH-L2-Gem has the same composition as YNH-L2-Gem except that it contains a scrambled peptide of sequence YDPS(Hsr)A(Nle)YSPSVK and it was synthesized using the same general scheme. Analytical data relative to critical intermediates and final compounds are reported as Supplementary Figure S4. Isothermal titration calorimetry data for YNH-L2-GEM against (**B**) EphA2 and (**C**) EphA3 LBD ligand binding domains are reported. For the binding between EphA2 LBD and YNH-L2-GEM the data revealed a Kd = 3.8 μM, ΔH = −16 Kcal/mol, −TΔS = −9.1 Kcal/mol. No appreciable binding was detected between YNH-L2-GEM and the EphA3 LBD (∼50% sequence identity with the EphA2 LBD). Similar data were obtained with 123B9-L2-GEM (Supplementary Figure S1).

The synthesis of YNH-L2-Gem (YDH-L2-Gem) and 123B9-L2-Gem followed the general scheme shown in Figure 1. The final drug conjugates were obtained by coupling the corresponding YNH, YDH or 123B9 motifs (Supplementary Figure S1) to the synthesized azidohexanoyl gemcitabine (Figure 1; Supplementary Figure S4). Briefly, a stirred solution of azidohexanoyl-gemcitabine (0.097 mmol) containing the corresponding EphA2 targeting motif (0.105 mmol) in DMSO-water (4:1, 3.0 mL) was added to CuSO_4_ (1.0 M, 50 μL) and sodium ascorbate (1.0 M, 50 μL) and continually stirred for another 2 days. The product was purified on a reverse phase C-18 column by HPLC with a gradient of 10–90% acetonitrile-water to give the title compounds as white powder.

To further verify the binding affinity and selectivity of the resulting conjugates for the EphA2 ligand binding domain (LBD), we expressed and purified EphA2 and EphA4 ligand binding domains (EphA2-LBD and EphA4-LBD). These proteins were dissolved to a final concentration of 100 μM in 50 mM phosphate buffer (pH = 6.5), containing 100 mM NaCl. Isothermal Titration Calorimetry (ITC) measurements under these experimental conditions revealed that YNH-L2-Gem and 123B9-L2-Gem bound to EphA2 with Kd values of 3.8 μM and 2.3 μM, respectively (Figure 1; Supplementary Figure S1). In contrast, no appreciable binding was observed for the scrambled YDH-motif to the EphA2-LBD or for any of the agents for the EphA3-LBD (Figure 1; Supplementary Figure S1). These data are consistent with our previous studies with unconjugated peptides and related Paclitaxel conjugates [28−31].

### Pancreatic cancer cell lines and human pancreatic cancer tissue contain elevated EphA2

**Figure 2:**
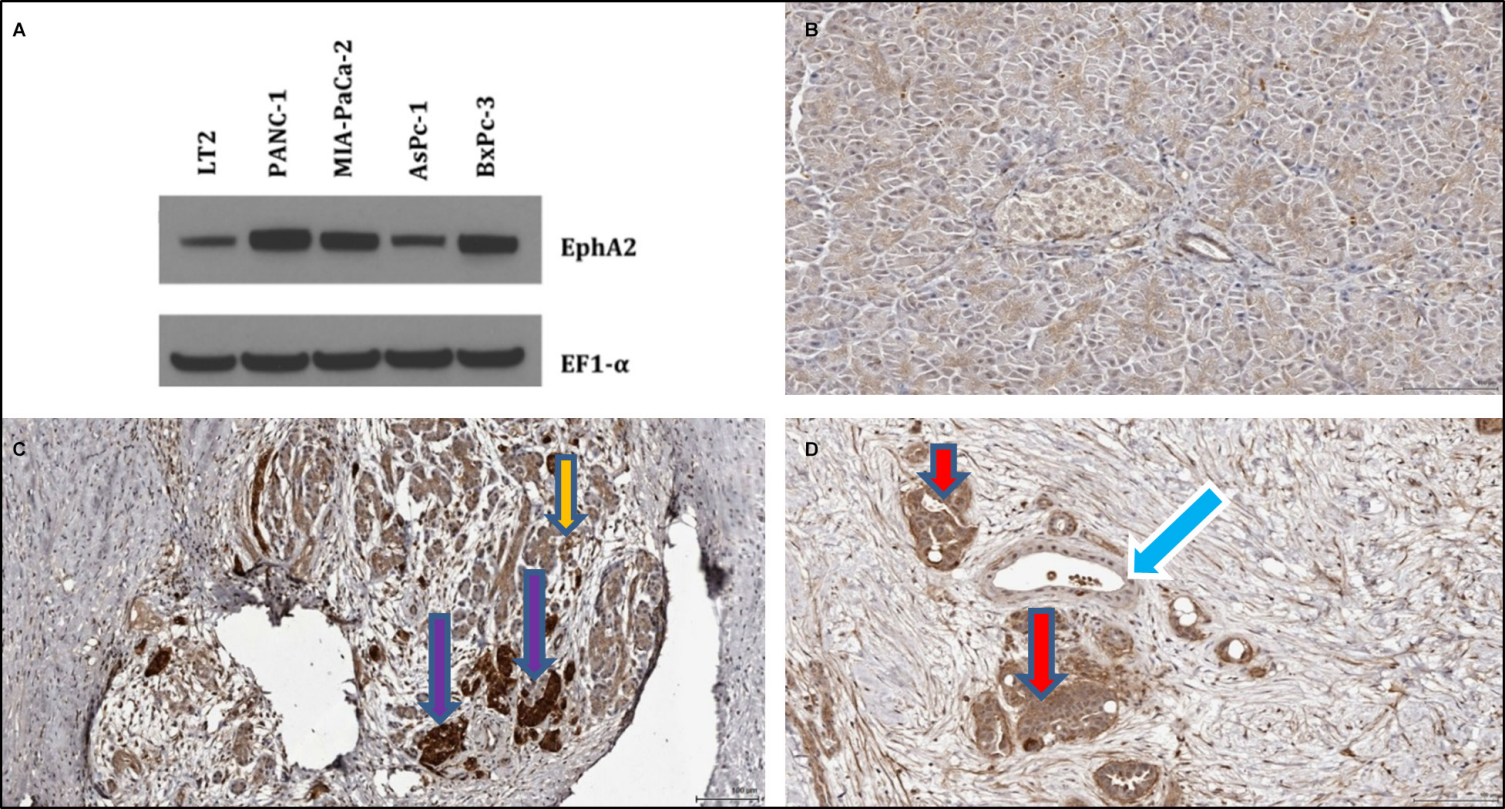
Expression pattern of EphA2 in cancer cells and pancreatic tissues (**A**) Western blotting of normal pancreatic fibroblasts (LT2) and pancreatic cancer cells (Panc-1, MIA-PaCa-2, AsPc-1, BxPc-3). Immunostaining of (**B**) normal pancreatic cancer tissue, (**C**) chronic pancreatitis tissue, and (**D**) pancreatic ductal adenocarcinoma tissue. EphA2 antibody appears to stain the surviving acinar tissue of chronic pancreatitis tissue (panel C), indicated by the orange arrow. In addition, some unknown tissue with islets characteristic morphology with high intensity within stroma of CP tissue is stained, as indicated by violet arrows (panel C). In the pancreatic ductal adenocarcinoma tissue, the EphA2 antibody stains cancer glands (red arrow, panel D) but not normal appearing tissues and ducts. Fibroblast cells also stains slightly. Normal ducts indicated by blue arrow.

Analysis of EphA2 expression in a variety of pancreatic cancer cell lines, including MIA PaCa-2, PANC-1, BxPC-3, and AsPC-1 cells (ATCC) and normal immortal LT2 cells (Millipore) was performed using western blotting experiments probed with a primary antibody for the EphA2 receptor (Cell Signaling, used at 1:1,000 dilution). Representative data obtained with 5 × 10^5^ cells are reported in Figure 2A. Overexpression of the EphA2 receptor appeared evident in all cell lines tested. Among these, the MIA-PaCa-2 cell line was chosen for subsequent xenograft studies.

Normal human pancreatic tissue, chronic pancreatitis tissue, and human pancreatic ductal adenocarcinoma tissue [34, 35] were also probed for EphA2 expression using a rabbit anti-EphA2 (ab78002, abcam, 1/200). We observed that the EphA2 antibody did not appear to stain normal tissue (Figure 2B) except perhaps for some slight staining around the center of some acini. In chronic pancreatitis tissue, we found EphA2 staining in surviving acinar tissue, and also in some unknown tissue with characteristic islets morphology (Figure 2C). Staining of the EphA2 antibody in the human pancreatic adenocarcinoma tissue was evident in cancerous, but not in normal ducts and also in some fibroblast cells within stroma (Figure 2D).

### EphA2-targeting-gemcitabine conjugates are more effective than gemcitabine as single agents in xenografts of pancreatic cancer

MIA PaCa-2-luc cells were used to create bilateral subcutaneous xenografts in athymic nude mice. Western blotting data showed that this pancreatic cancer cell line highly expresses EphA2 (Figure 2A). Cells were injected into each flank and then allowed to grow for approximately 1 week. At this point, mice were imaged using BLI and treatment was subsequently initiated. Mice were divided into 4 groups (PBS, Gemcitabine, YDH-L2-Gemcitabine, YNH-L2-Gemcitabine) with 9 mice/group. YDH-L2-Gemcitabine is a scrambled control, where the peptide attached to Gemcitabine should not be specific for EphA2 and, therefore, should not bind to the receptor. Animals were treated 2 times per week via tail vein injection for 4 weeks. A dose of 10 mg/kg Gemcitabine was used along with equimolar doses of YDH-L2-Gemcitabine and YNH-L2-Gemcitabine. At the end of 4 weeks, animals were again imaged and treatment was ended.

**Figure 3:**
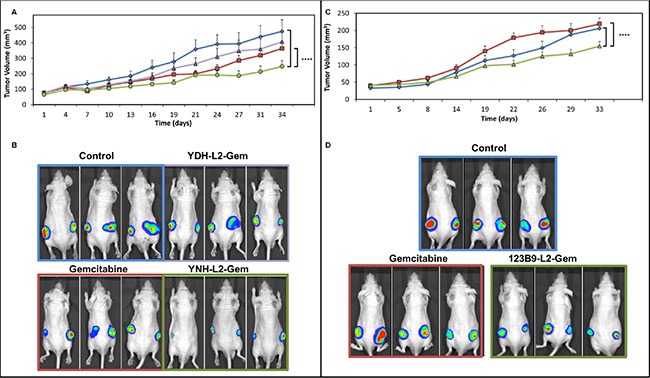
In vivo efficacy studies with gemcitabine and its EphA2-targetting-gemcitabne conjugates Each treated mice received the equivalent of 10 mg/Kg of Gemcitabine. (**A**) YNH-L2-Gemcitabine inhibits tumor growth *in vivo* to a greater extent as compared to Gemcitabine. Tumor volume is reported as calculated by caliper tumor measurement (blue diamonds, control; red squares, Gemcitabine; green circles, YNH-L2-GEM; violet triangles, YDH-L2-GEM); for statistical comparisons we used the two-way Anova analyses (GraphPad), ****p < 0.0001. (**B**) Bioluminescent tumor images taken for representative mice in each group at Day 34. Exposure time = 0.5 seconds. (**C**) *In vivo* efficacy studies with Gemcitabine and its 123B9-L2-Gem conjugate (blue diamonds, control; red squares, Gemcitabine; green circles, 123B9-L2-GEM) (**D**) Bioluminescent tumor images taken for representative mice in each group at day 33. Exposure time = 0.5 seconds.

As expected, YDH-L2-Gemcitabine did not have significant effects on tumor growth. Gemcitabine had a modest effect on tumor growth, though YNH-L2-Gemcitabine showed the greatest inhibition of tumor growth of all groups evaluated (Figure 3A). This can be observed both through tumor measurement and by BLI (Figure 3B). No negative side effects of the drugs were observed and the mouse weight remained consistent throughout the study. 3 animals per group were sacrificed at the end of this study and tumors were excised and fixed in formalin for future analyses. The remaining mice were sacrificed only when they reached a moribund status. From this study we observed that both Gemcitabine and YNH-L2-Gemcitabine treated mice displayed a significant increase in the median survival time compared to untreated mice (Supplementary Figure S2).

Similarly, in a separate experiment we compared the efficacy of equimolar doses of Gemcitabine and 123B9-L2-Gem (Figure 3C, 3D). As in the case of YNH-L2-Gem, we found that 123B9-L2-Gem was significantly more effective than gemcitabine alone in suppressing tumor growth. However, the median survival time of 123B9-L2-Gem treated mice was significantly longer than both untreated and Gemcitabine treated groups (Supplementary Figure S2). This again suggests that the targeted delivery approach may result in an accumulation of the drug in the EphA2 expressing tumor cells (See also Supplementary Figure S5).

## DISCUSSION

Though not overly efficacious, chemotherapy remains the mainstay of pancreatic cancer therapy. There is a vital need to develop novel therapies that provide greater clinical benefit to patients without undue toxicity. A Phase 3 clinical study in patients with metastatic pancreatic cancer evaluated nab-Paclitaxel (Abraxane) + Gemcitabine versus Gemcitabine alone in a total of 861 patients. The median survival was 8.5 months in the nab-paclitaxel + Gemcitabine group vs. 6.7 months in the Gemcitabine group. The survival rate at 1 year was 35% vs. 22%; 9% vs. 4% at 2 years. The response rate was 23% for combination group and 7% for Gemcitabine alone. Toxicities included neutropenia, fatigue, and neuropathy [36]. These results encouraged the FDA to approve the combination of Gemcitabine and nab-Paclitaxel for metastatic pancreatic cancer.

Accordingly, in preliminary experiments we investigated whether the efficacy of Gemcitabine against pancreatic cancer as a single agent could be improved using our recently developed targeted delivery strategy. First, we developed drugs conjugates that are capable of specifically targeting the EphA2 receptor, which is overexpressed on the surface of pancreatic cancer cells. Initial studies used Gemcitabine linked to the YNH peptide and its scrambled control YDH-L2-Gem (Figure 1). Similarly, we synthesized 123B9-L2-Gem, in which the terminal tyrosine of the EphA2 targeting peptide, which is essential to specific EphA2 binding, was replaced with a bioisoster, resulting in increased stability and longer half-life *in vivo* of the agent [30]. Our previous studies demonstrated that YSA-L1-palitaxel conjugates, when administered in prostate cancer (PC3) xenografts bearing mice resulted in the accumulation of the drug in the tumor, using extraction and LC/MS analysis [28]. Here, we chose the L2 linker as we recently studied in details the degradation of these conjugates in plasma [29] and *in vivo* [30] and concluded that this linker is stable and long lived compared to previously proposed linker. In particular, the hydrolysis of the L2 linker and concomitant delivery of the free payload started to occur after 8 hr incubation of the conjugate with rat plasma [29]. To further corroborate these previous findings we conjugated 123B9 or a scrambled version with a near infrared dye (Supplementary Figure S5) for *in vivo* imaging studies. Similar to what previously found using LC/MS analysis [28], we observed increased fluorescence in the tumor site in mice bearing PC3 xenografts compared to mice that received a scrambled-NIR conjugate (Supplementary Figure S5) further supporting that the proposed targeting agents can direct the payload at EphA2 rich tumor sites. Hence, to investigate whether these findings in prostate cancer could be translated to pancreatic cancers, we subsequently evaluated the expression of the EphA2 receptor in a variety of pancreatic cancer cell lines. Most cell lines tested exhibited elevated EphA2 levels and among these we selected the MIA PaCa-2 cell line for subsequent *in vivo* studies (Figure 2A). In addition, and perhaps of most relevance, EphA2 overexpression appeared evident in diseased versus normal pancreatic tissues (Figure 2B–2D). Hence, we preliminarily evaluated the ability of these novel targeting agents to inhibit tumor growth in pancreatic cancer xenograft studies using the MIA PaCa-2 cell line. Gemcitabine as a single agent at the chosen dose and regimen did not exhibit significant efficacy compared to mice treated with vehicle only as control, as indicated by tumor growth (Figure 3). On the contrary, significant efficacy was observed in the xenograft studies in mice receiving either YNH-L2-Gem or 123B9-L2-Gem (Figure 3). In addition, a significant increase in survival time was observed in mice treated with 123B9-L2-Gem compared to both Gemcitabine treated and untreated groups.

Our preliminary studies on the expression patterns in pancreatic tissues from human biopsies clearly indicated elevated EphA2 in the diseased versus normal pancreatic tissue (Figure 2B–2D). The EphA2 antibody did n't appear to stain normal pancreatic tissue, whereas staining of surviving acinar tissue and the stroma of chronic pancreatitis tissue was evident. In addition, in human pancreatic ductal adenocarcinoma, the EphA2 antibody clearly stained cancerous ducts and fibroblast cells within stroma. These data strongly suggest that our targeted delivery approach may be even more efficacious in the natural micro-environment of the pancreas. In conclusion, albeit at preliminary stages, our data support the use of these novel EphA2-targeting drug conjugates suggesting that they may provide an exciting new strategy for pancreatic cancer drug development. Overall, these studies provide encouraging evidence that conjugation of chemotherapy to our EphA2 targeting agents may have potential to improve clinical outcome in pancreatic cancer patients. Additional studies on 123B9-L2-Gem and/or YNH-L2-Gem as single agents or in combination with nab-paclitaxel (Abraxane), perhaps using more complex genetically modified models of pancreatic cancers with an intact immune system that recapitulate pancreatic cancer development in patients, are warranted to further evaluate these exciting perspectives.

## MATERIALS AND METHODS

### Synthesis and characterization of EphA2 targeting agents conjugated with gemcitabine

All linear peptides and peptide mimetics were assembled using standard Fmoc peptide synthesis protocol with the Rink amide resin on 433A Peptide Synthesizer (Applied Bio-Systems). In general, in each coupling reaction, approximately 10 equivalents of Fmoc-amino acid, 0.45 M solution of HOBt/HBTU (9 equiv) in DMF, 2 M solution of DIEA in NMP were used, for about 9 minutes. Fmoc de-protection was performed with 20% piperidine in DMF for 10 min. Each peptide was subsequently cleaved from the resin and all protecting groups removed by exposure to a 94% TFA, 2 % water, 2% tri-isopropylsilane, 2% phenol mixture for approximately 3 h. The TFA mixture was subsequently removed under reduced pressure and the peptides were precipitated in diethyl ether, centrifuged, and washed with diethyl ether prior to drying in high vacuum. The crude peptides were purified by preparative reverse phase HPLC. The final compounds were characterized by NMR and MALDI-Mass. All compounds were of > 95% purity. EphA2-LBD and EphA3-LDB were expressed as reported recently [30].

Isothermal Titration Calorimetry (ITC) measurements were obtained with Model ITC200 from Microcal/GE Life Sciences. For *in vivo* studies, all drugs were diluted in 10% Tween-80, 10% DMSO, and 80% PBS.

### EphA2 expression levels in human cell lines and tissues

MIA PaCa-2, PANC-1, BxPC-3, and AsPC-1 cells were all obtained from the American Type Culture Collection (ATCC). LT2 immortal normal mesenchymal cells were purchased from Millipore. MIA PaCa-2 and PANC-1 were maintained in DMEM plus 10% FBS. BxPC-3 and AsPC-1 cells were maintained in RPMI plus 10% FBS. LT2 cells were maintained with media according to distributor's instructions. Cell lines were expanded and cryopreserved at early passages and new vials were thawed and used for experiments approximately every 3 months. 5 × 10^5^ cells were plated in 6-cm dishes and treated as described. After 48 hours, whole cell lysates were prepared and western blotting analysis was carried out as previously described [37]. Primary antibodies used for these studies were EphA2 (1:1,000, Cell Signaling) and EF1-α (1:5,000, Sigma). Representative data are reported in Figure 2A.

Normal pancreatic tissue (N-pancreas) chronic pancreatitis tissue (CP), and Pancreatic ductal adenocarcinoma (PDAC) tissue were obtained from an IRB approved protocol at Cedar-Sinai Medical Center (34086) and probed for EphA2 expression using a rabbit anti-EphA2, (ab78002, abcam, 1/200). Representative data are reported in Figure 2B–2D.

### Subcutaneous xenograft studies

5 × 10^6^ MIA PaCa-2-luciferase cells were used to establish bilateral subcutaneous tumors on the flanks of 8–10 week old male athymic nude mice. Studies were done as previously described [33]. Treatment began when tumors reached a palpable size (∼50–100 mm^3^) with 9 mice per group. All drugs were administered by tail vein injection twice per week for 4 weeks, for a total of 8 injections. Gemcitabine was given at a dose of 10 mg/kg and all Gemcitabine derivatives were given at equimolar doses to 10 mg/kg Gemcitabine (hence we injected ∼70 mg/Kg of the conjugated having a MW ∼7 times greater than Gemcitabine), also via tail vein injection. BLI measurements were performed at the beginning and end of the study. During imaging, mice were placed in the imaging chamber and maintained with 2% isoflurane gas anesthesia at a flow rate of approximately 0.5–1 L/min per mouse. Anesthetized mice were injected IP with 150 mg/kg body weight D-Luciferin (Xenogen Corporation, Alameda, CA). After approximately 10 min, mice were imaged using a charge-coupled-device (CCD) camera coupled to the Xenogen *in vivo* imaging (IVIS) imaging system (Caliper Life Sciences, Inc., Hopkinton, MA).

Tumors were measured twice per week using calipers. For *in vivo* studies, data shown are the mean + SEM and for statistical comparisons we used the two-way Anova analyses (GraphPad). After their last injection, mice were kept for 1 additional week to monitor tumor growth and imaged at the end of that week. The mice were then kept to monitor for the effects of treatment on survival. Mice were kept until reaching a moribund status and they were then sacrificed at that time.

## SUPPLEMENTARY MATERIALS FIGURES


