# Supplementary material for: Therapy of pancreatic cancer via an EphA2 receptor-targeted delivery of gemcitabine
Source: Oncotarget. 2016 Mar 5;7(13):17103–10. doi: 10.18632/oncotarget.7931 (PMC4941374; doi:10.18632/oncotarget.7931)
Supplement: Supplementary file 1 [file oncotarget-07-17103-s001.pdf]

Therapy of pancreatic cancer via an EphA2 receptor-targeted delivery of gemcitabine

Supplementary Materials

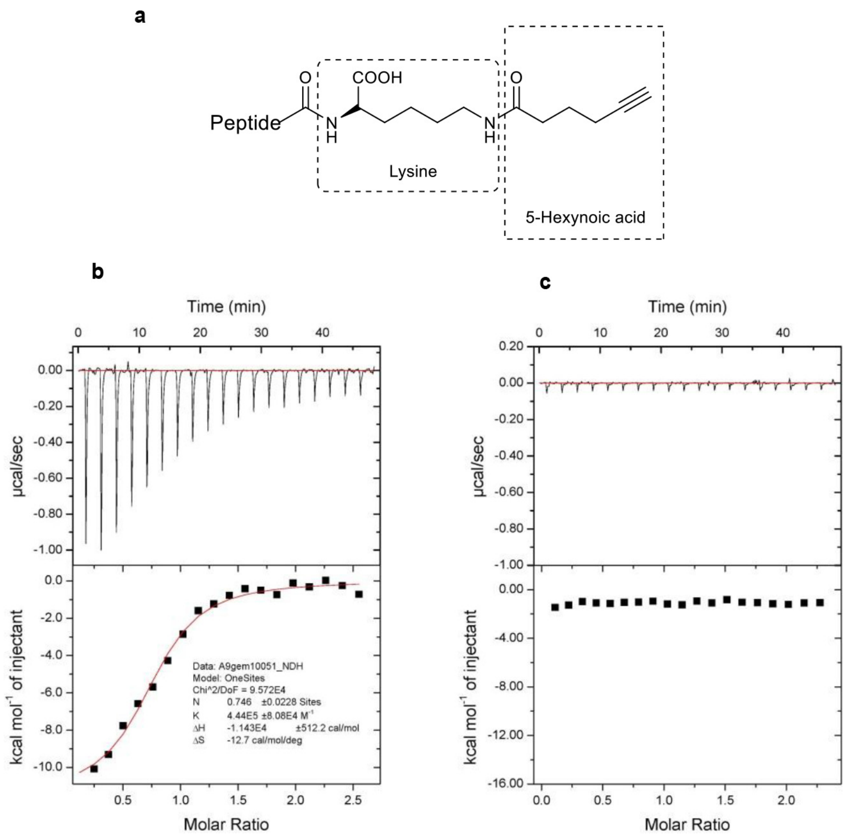

Supplementary Figure S1: (**A**) General chemical structure of the YNH-motif, 123B9-motif, or YDH-motif. A 5-hexynoic acid lysine linker is included at the C-terminal of each peptide subsequent use is the click–chemistry based generation of the final conjugates (Figure 1). The motifs have been obtained as we described recently (30). Isothermal titration calorimetry data for 123B9-L2-GEM against (**B**) EphA2 and (**C**) EphA3 LBD ligand binding domains are reported. For the binding between EphA2 LBD and 123B9-L2-GEM (B) the data revealed a Kd = 2.3 µM, ΔH = −11 Kcal/mol, −TΔS = −3.7 Kcal/mol. (**C**) No appreciable binding was detected between 123B9-L2-GEM and the EphA3 LBD (~50% sequence identity with the EphA2 LBD).

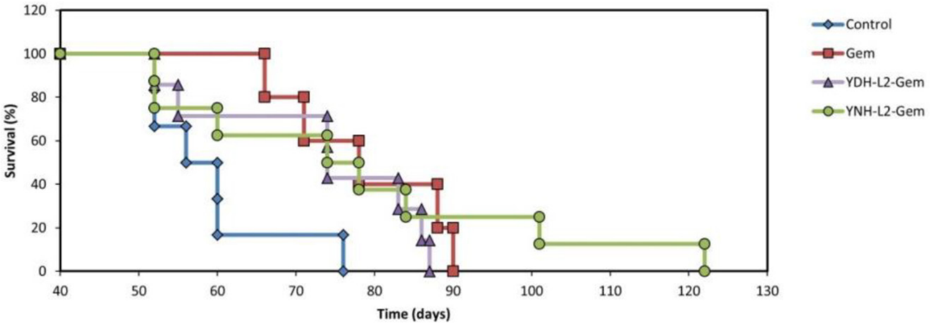

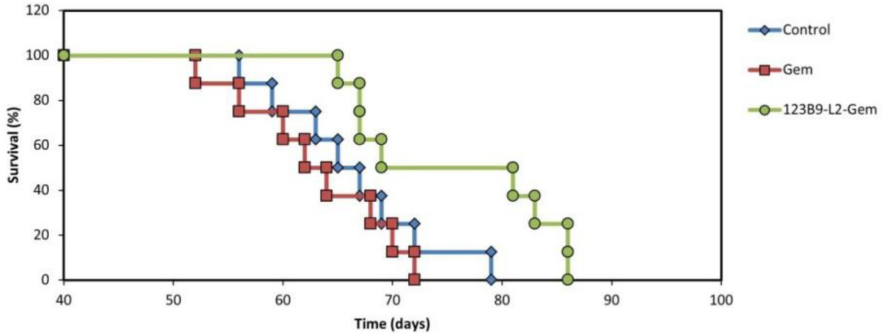

Supplementary Figure S2: YNH-L2-Gem (top) and 123B9-L2-Gem (bottom) prolong survival in xenograft models of pancreatic cancer. Kaplan-Meier curve showing survival in days.

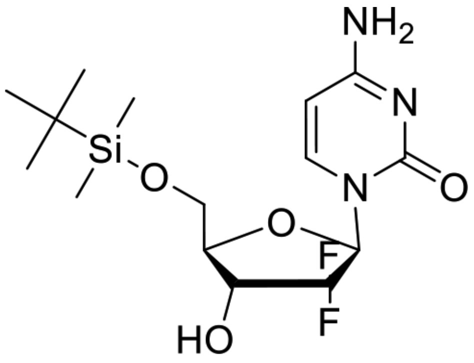

Preparation of 5′-O-TBS-gemcitabine (1)

To a stirred mixture of gemcitabine (1.50 g, 5.66 mmol) and imidazole (503 mg, 7.40 mmol) in DMF (5.0 mL) was added TBSCl (1.12 g, 7.40 mmol) at room temperature. The resulting mixture was stirred overnight and poured into EtOAc (50 mL). The organic phase was washed with brine (50 mL × 3), dried over Na_2SO_4, filtered, evaporated to dryness. The residue was purified by silica gel chromatography, eluted with MeOH/DCM (1–10%) to provide the desired product 1 (1.56 g, 72%).

^{1}H NMR (DMSO-d_6, 600 MHz): δ 7.63 (d, J = 8.4 Hz, 1 H), 7.40 (d, J = 7.7 Hz, 2 H), 6.31 (d, J = 7.7 Hz, 2 H), 6.13 (s, 1 H), 5.75 (d, J = 8.4 Hz, 1 H), 4.11 (s, 1 H), 3.95 (d, J = 12.0 Hz, 1 H), 3.86 (d, J = 10.8 Hz, 1 H), 3.82 (d, J = 14.0 Hz, 1 H), 0.89 (s, 9 H), 0.08 (s, 6 H);

^{13}C NMR ($CDCl_3$, 150 MHz): δ 165.99, 154.97, 140.31, 123.38 (t, J = 257.0 Hz), 94.92, 83.85 (t, J = 32.8 Hz), 80.14, 68.60 (t, J = 22.7 Hz), 61.11, 26.15, 18.42, −2.51;

ESI-MS (*m/z*): $[M + H]^+$ calcd for $C_{15}H_{26}F_2N_3O_4Si$, 378; found, 378;

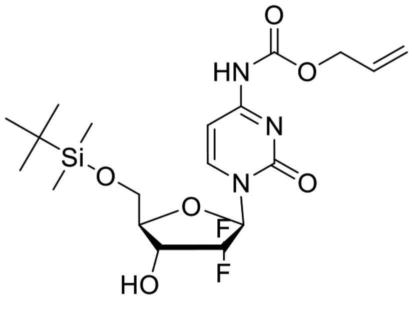

Preparation of 5′-O-TBS-4-N-Alloc-gemcitabine (2)

To a stirred mixture of compound 1 (1.56 g, 4.13 mmol) and Et_3N (1.5 mL, 10.8 mmol) in anhydrous THF (8.0 mL) was added diallyl pyrocarbonate, (1.15 g, 6.19 mmol) at room temperature. The resulting mixture was

stirred overnight and evaporated to dryness. The residue was purified by silica gel chromatography, eluted with MeOH/DCM (1–10%) to provide the desired product 2 (1.53 g, 81%).

^{1}H NMR (DMSO-d_6, 600 MHz): δ 7.58 (d, J = 8.4 Hz, 1 H), 7.46 (d, J = 7.7 Hz, 2 H), 6.25 (s, 1 H), 5.96 (m, 1 H), 5.80 (d, J = 8.40 Hz, 1 H), 5.38 (d, J = 16.2 Hz, 1 H), 5.30 (d, J = 10.8 Hz, 1 H), 4.70 (d, J = 7.80 Hz, 1 H), 4.25 (s, 1 H), 3.95 (d, J = 12.0 Hz, 1 H), 3.89 (d, J = 10.8 Hz, 1 H), 3.80 (s, 1 H), 0.88 (s, 9 H), 0.08 (s, 6H);

^{13}CNMR (DMSO-d_6, 150MHz): δ 166.08, 154.75, 153.36, 141.17, 131.90, 123.37 (t, J = 257.0 Hz), 122.02, 119.43, 95.23, 83.41(t, J = 32.8 Hz), 77.96, 73.54 (t, J=22.7 Hz), 69.47, 61.60, 26.30, 18.28, −2.51;

ESI-MS (*m/z*): $[M+H]^+$ calcd for $C_{19}H_{30}F_2N_3O_6Si$, 462; found, 462;

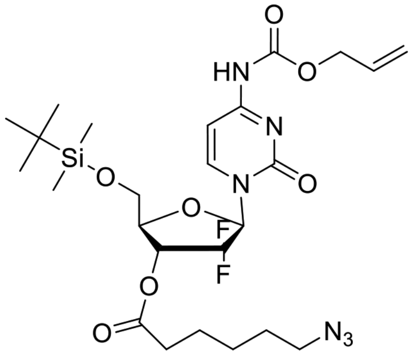

Preparation of 3′-O –(6-azidohexanoyl)-5′-O-TBS-4-N-Alloc-gemcitabine (3)

To a solution of compound 2 (1.53g, 3.31 mmol) in dichloromethane (DCM, 10 mL) was added EDCI (1.26 g, 6.63 mmol), DMAP (848 mg, 6.63 mmol), and 6-azidohexanoic acid (1.03g, 6.63 mmol) at room temperature and stirred for 3 h. After removal of the solvents, the residue was partitioned between EtOAc (50 mL) and brine (50 mL). The water phase was extracted with EtOAc (50 mL × 2), and the organic phases were combined, dried over Na_2SO_4, filtered, and evaporated to dryness. The residue was purified on a flash silica gel column eluted with ethyl acetate-hexane (10–70%) to give the title compound (1.89 g, 95%).

^{1}H NMR ($CDCl_3$, 600 MHz): δ 8.04 (d, J = 8.4 Hz, 1 H), 7.51 (d, J = 7.7Hz, 1 H), 7.27(s, 1 H), 6.44 (m, 1 H), 5.97 (m, 1 H), 5.42 (d, J = 12.0Hz, 1 H), 5.31 (m, 2H), 4.71 (d, J = 7.2Hz, 2 H), 4.24 (s, 1 H), 4.02 (d, J = 12.0 Hz, 1 H), 3.78 (m, 2 H), 3.30 (t, J = 7.2Hz, 2 H), 2.42 (t, J = 7.2Hz, 2 H), 1.74 (m, 4 H), 1.46 (m, 2 H), 0.95 (s, 9 H), 0.13 (s, 6 H);

^{13}CNMR ($CDCl_3$, 150 MHz): δ 178.81, 173.63, 154.35, 153.25, 144.74, 130.56, 121.09 (t, J = 257.0 Hz), 119.89, 96.93, 84.33(t, J = 32.8 Hz), 79.69, 72.52 (t, J = 22.7 Hz), 69.66, 60.70, 51.15, 37.12, 34.14, 28.52, 26.22, 25.83, 24.36, 24.06, 18.23, −2.51;

ESI-MS (*m/z*): $[M+H]^+$ calcd for $C_{25}H_{39}F_2N_6O_7Si$, 601; found, 601;

Preparation of 3′-O –(6-azidohexanoyl) -4-N-Alloc-gemcitabine (4) and 3′-O –(6-azidohexanoyl)-gemcitabine (5)

To a solution of compound 3 (1.50 g, 2.50 mmol) was added tetrabutylammonium fluoride solution (TBAF, 1.0 M, 5.0 mL) at room temperature and stirred for 30 min. After removal of solvent, the residue was purified on a flash silica gel column eluted with MeOH:DCM (5–10%) to give compound 4 (672 mg, 55%) and compound 5 (437 mg, 43%).

3′-O –(6-azidohexanoyl) -4-N-Alloc-gemcitabine (4)

^{1}H NMR (DMSO-d_6, 600 MHz): δ 11.03 (s,1 H), 8.01 (d, J = 8.4 Hz, 1 H), 7.31(d, J = 8.4 Hz, 1 H), 6.29 (s, 1 H), 5.96 (m, 1 H), 5.36 (d, J = 12.0 Hz, 1 H), 5.25 (d, J = 10.8 Hz, 1 H), 4.64 (d, J = 7.8 Hz, 1 H), 4.44 (m, 2 H), 4.23 (m, 1 H), 4.13 (s, 1 H), 3.78 (s, 1 H), 3.30 (t, J = 7.2 Hz, 2 H), 2.42 (t,J = 7.2 Hz, 2 H), 1.56 (m, 4 H), 1.31 (m, 2 H);

^{13}CNMR (DMSO-d_6, 150 MHz): δ 174.37, 163.29, 154.49, 154.38, 145.16, 123.36 (t, J = 257.0 Hz), 96.32, 84.52 (t, J = 32.8 Hz), 81.40, 68.74 (t, J = 22.7 Hz), 59.17, 50.87, 36.64, 28.38, 26.01, 24.29;

ESI-MS (*m/z*): $[M + H]^+$ calcd for $C_{19}H_{25}F_2N_6O_7$, 487; found, 487;

3′-O –(6-azidohexanoyl)-gemcitabine (5)

^{1}H NMR (DMSO-d_6, 600 MHz): δ 11.00 (s,1 H), 8.24 (d, J = 8.4 Hz, 1 H), 7.29 (d, J = 8.4 Hz, 1 H), 6.32 (d, J = 8.4 Hz, 1 H), 6.18 (t, J = 7.2 Hz, 1 H), 5.30 (d, J = 7.2 Hz, 1 H), 4.18 (m, 1 H), 3.89 (m, 1 H), 3.81 (m, 1 H), 3.65 (m, 1 H), 3.30 (t, J = 7.2 Hz, 2H), 2.41 (t, J = 7.2 Hz, 2 H), 1.53 (m, 4 H), 1.31 (m, 2H);

^{13}CNMR (DMSO-d_6, 150 MHz): δ 174.37, 163.29, 154.62, 145.16, 123.36 (t, J = 257.0 Hz), 96.32, 84.52 (t, J = 32.8 Hz), 81.40, 68.74 (t, J = 22.7 Hz), 60.17, 50.98, 36.64, 28.38, 26.01, 24.29;

ESI-MS (*m/z*): $[M+H]^+$ calcd for $C_{15}H_{21}F_2N_6O_5$, 403; found, 403;

Converted compound 4 into compound 5

To a stirred solution of compound 4 (500 mg, 1.03 mmol) in THF (5.0 mL) was added $PdCl_2(Ph_3P)_2$ (91 mg, 0.13 mmol) and HOAc (200 uL) at 0°C, and followed by addition of tributyltin hydride (359 mg, 1.23 mmol). The resulting mixture was stirred for 2 h. The solvent was removed, and the residue was purified on a flash silica gel column eluted with MeOH:DCM (5–10%) to give compound 5 (345 mg, 83%).

Preparation of 123B9-L2-GEM (6)

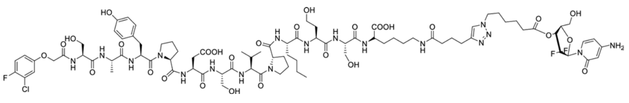

To a stirred solution of compound 5 (60 mg, 0.148 mmol) and 123B9-motif (230 mg, 0.146 mmol) in DMF-water (4: 1, 3.0 mL) was added the solutions of $CuSO_4$ (1.0 M, 50 µL) and sodium ascorbate (1.0 M, 50 µL) and continually stirred for another 2 days. The product was purified on the reverse phase C-18 column by HPLC with a gradient of 10–90% acetonitrile-water to give the title compound as the trifuoroacetic acid salt (158 mg, 55%).

^{1}H NMR (600 MHz, DMSO-d_6): δ 7.70−8.23 (m, 15 H), 7.62 (d, *J* = 7.8 Hz, 1 H), 6.90−7.45 (m, 9 H), 6.64 (d, *J* = 7.8 Hz, 2 H), 6.21 (s, 1 H), 4.00− 4.70 (m, 17 H), 3.20−3.90 (m, 15 H),), 3.05 (m, 2 H), 2.91 (m, 1 H), 2.50−2.85 (m, 7 H), 2.41 (t, *J* = 7.2 Hz, 2 H), 2.05−2.30 (m, 4 H), 1.45−2.05 (m, 18 H), 1.24−1.48 (m, 10 H), 1.11 (d, *J* = 7.2 Hz, 3 H), 0.89 (d, *J* = 7.2 Hz, 3 H), 0.88 (d, *J* = 7.2 Hz, 3 H); 0.83 (t, *J* = 7.2 Hz, 6 H);

^{13}C NMR (150 MHz, DMSO-d_6): δ 174.37, 174.20, 172.45, 172.32, 172.23, 172.12, 170.73, 170.61, 170.51, 170.26, 170.12, 169.98, 169.69, 167.63, 156.20, 154.72, 154.65, 153.35 (t, J_{CF} = 245 Hz), 151.76, 146.80, 145.25, 130.64, 128.11, 123.12 (t, J = Hz), 120.17, 120.04, 118.51, 117.52, 116.89, 115.62, 115.58, 115.36, 96.36, 84.74, 81.39, 68.74 (t, J = Hz), 67.73, 62.14, 62.03, 60.19, 59.65, 59.16, 57.96, 56.18, 55.45, 55.26, 54.73, 53.24, 53.01, 50.80, 50.75, 49.96, 49.47, 48.61, 48.56, 47.51, 47.22, 38.75, 36.58, 36.15, 36.07, 35.26, 33.83, 31.85, 31.51, 30.45, 29.86, 29.34, 29.24, 28.39, 27.76, 25.82, 25.64, 25.04, 24.90, 24.82, 24.26, 23.26, 22.33, 19.75, 19.54, 18.33, 14.21;

MS (MALDI-TOF, *m/z*): [M + H]$^+$ calcd for $C_{86}H_{121}ClF_3N_{18}O_{28,}$ 1946; found, 1946.

Preparation of YNH-L2-GEM (7)

The title compound was prepared using a method similar to that described for 123B9-L2-GEM (6)

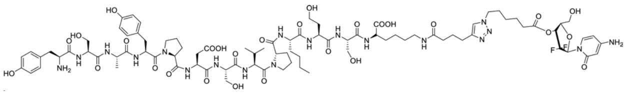

^{1}H NMR (600 MHz, DMSO-d_6): δ 11.0 (s, 1 H), 8.68 (d, *J* = 7.8 Hz, 1 H), 7.90−8.50 (m, 9 H), 7.50−7.90 (m, 7 H), 7.27 (d, *J* = 7.8 Hz, 1 H), 7.21 (s, 1 H), 7.06 (m, 4 H), 6.69 (d, *J* = 7.8 Hz, 2 H),

6.64 (d, *J* = 7.8 Hz, 2 H), 6.17 (s, 1 H), 4.00−4.60 (m, 15 H), 3.20−4.00 (m, 13 H), 2.99 (m, 1 H), 2.91 (m, 1 H), 2.50 – 2.85 (m, 9 H), 2.41 (t, *J* = 7.2 Hz, 2 H), 2.05–2.30 (m, 4 H), 1.45−2.05 (m, 18 H), 1.24−1.48 (m, 10 H), 1.15 (d, *J* = 7.2 Hz, 3 H), 0.89 (d, *J* = 7.2 Hz, 3 H), 0.85 (d, *J* = 7.2 Hz, 3 H); 0.84 (t, *J* = 7.2 Hz, 6 H);

^{13}C NMR (150 MHz, DMSO-d_6): δ 174.71, 174.28, 172.81, 172.64, 172.31, 172.16, 170.82, 170.81, 170.52, 170.41, 170.39, 169.90, 168.51, 163.21, 159.71, 159.62, 158.73, 158.55, 157.01, 156.21, 154.73, 146.33, 145.21, 130.93, 130.65, 128.11, 125.19, 123.19 (t, J = 159 Hz), 116.72, 115.31, 96.32, 84.71, 81.41, 68.73 (t, J = Hz), 65.64, 62.25, 62.05, 60.12, 59.59, 59.16, 57.96, 56.17, 55.41, 55.05, 53.96, 53.16, 52.99, 52.74, 50.88, 50.72, 49.40, 48.59, 48.11, 47.51, 47.13, 39.12, 38.75, 36.63, 36.13, 35.26, 32.25, 31.92, 31.54, 30.48, 29.88, 29.33, 28.41, 27.77, 27.61, 27.2, 26.01, 25.74, 25.65, 25.07, 24.90, 24.80, 24.15, 23.27, 22.31, 19.52, 18.67, 18.46, 14.26;

MS (MALDI-TOF, *m/z*): [M + H]$^+$ calcd for $C_{87}H_{126}F_2N_{19}O_{28,}$ 1923; found, 1923.

Figure S3 Synthetic procedures and analytical data relative to the preparation of compounds reported in Figure 1.

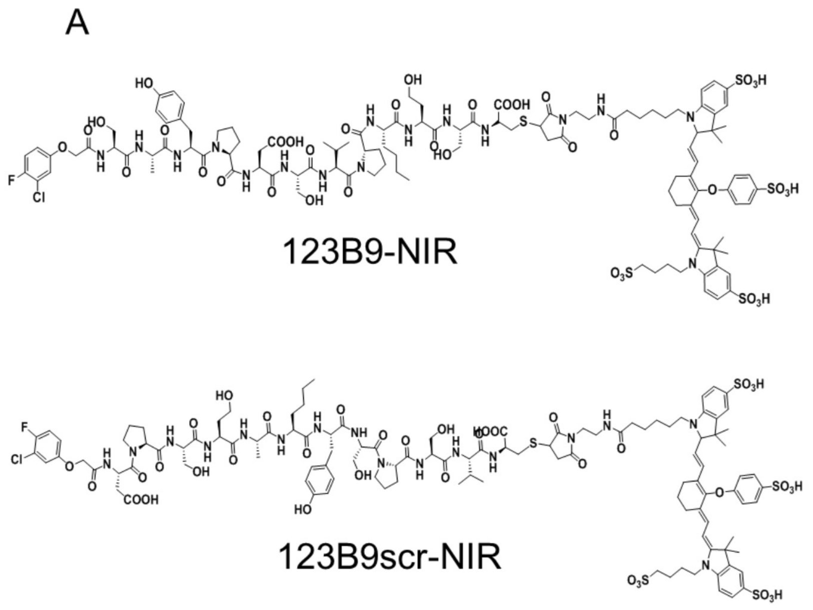

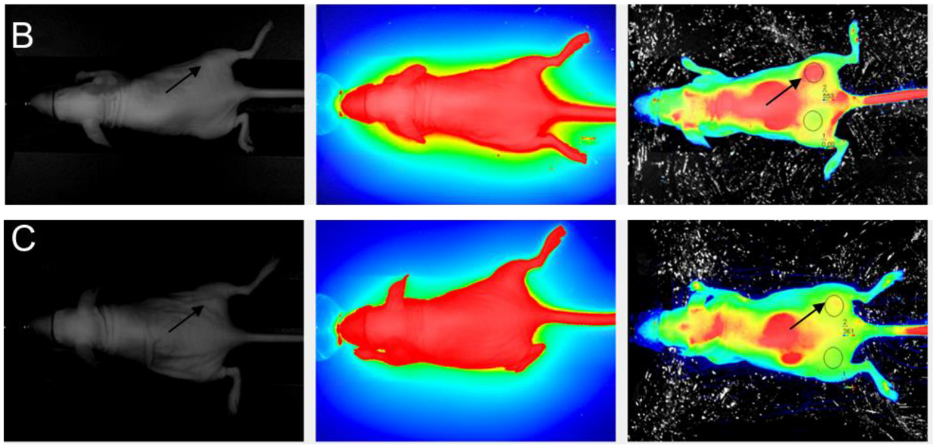

Supplementary Figure S3: 123B9 targeted tumor site *in vivo*. (**A**) We conjugated 123B9 or the reported scrambled equivalent to a near infra-red (NIR) dye for *in vivo* imaging. The NIR dye is routinely used in ensemble fluorescence imaging, because absorption, auto-fluorescence and scattering are all lower in biological cells and tissues at longer wavelengths. For visualization, the near infra-red dye 800 CW (NIRD800 CW) was conjugated to the SH group in a Cys side chain introduced at the C-terminal end of 123B9 (123B9-NIR). As a negative control, another agent was prepared in which the residue sequence of 123B9 was scrambled except for P-1 residue and subsequently conjugated to the NIRD800 CW to obtain a control 123B9scr-NIR. The chemical structures of these peptide-dye conjugates are reported. The agents were obtained using the same general synthetic methods used to obtain 123B9-Gem. *In vivo* imaging studies of 123B9-NIR (**B**) and the negative control 123B9scr-NIR (**C**) were carried out with mice bearing PC3 prostate cancer xenografts. Mice with subcutaneous tumor xenografts of comparable sizes implanted on the right flank (black arrows) were injected intravenously with either 123B9-NIR or 123B9scr-NIR (30 mg/Kg) and imaged prior to the injections, immediately after the injection and at 24 hours after injection. The accumulation of NIR fluorescence in the kidneys and heart was expected due to the relatively low molecular weight of 123B9. As shown, at 24 h post-injection of 123B9-NIR, a clear accumulation of NIR fluorescence was observed at the tumor site (panel B, black arrow), while no accumulation of fluorescence was found at the opposite flank. However, injection with the scrambled 123B9scr-NIR did not result in significant accumulation of fluorescence at either flanks including therefore at the tumor site (C).
